# Phytochrome A Mediates Blue-Light Enhancement of Second-Positive Phototropism in Arabidopsis

**DOI:** 10.3389/fpls.2016.00290

**Published:** 2016-03-11

**Authors:** Stuart Sullivan, Jaynee E. Hart, Patrick Rasch, Catriona H. Walker, John M. Christie

**Affiliations:** Institute of Molecular, Cell and Systems Biology, College of Medical, Veterinary and Life Sciences, University of GlasgowGlasgow, UK

**Keywords:** phototropism, blue light, red light, phototropin, phytochrome, phosphorylation, epidermis

## Abstract

Hypocotyl phototropism of etiolated Arabidopsis seedlings is primarily mediated by the blue-light receptor kinase phototropin 1 (phot1). Phot1-mediated curvature to continuous unilateral blue light irradiation (0.5 μmol m^−2^ s^−1^) is enhanced by overhead pre-treatment with red light (20 μmol m^−2^ s^−1^ for 15 min) through the action of phytochrome (phyA). Here, we show that pre-treatment with blue light is equally as effective in eliciting phototropic enhancement and is dependent on phyA. Although blue light pre-treatment was sufficient to activate early phot1 signaling events, phot1 autophosphorylation *in vivo* was not found to be saturated, as assessed by subsequently measuring phot1 kinase activity *in vitro*. However, enhancement effects by red and blue light pre-treatment were not observed at higher intensities of phototropic stimulation (10 μmol m^−2^ s^−1^). Phototropic enhancement by red and blue light pre-treatments to 0.5 μmol m^−2^ s^−1^ unilateral blue light irradiation was also lacking in transgenic Arabidopsis where *PHOT1* expression was restricted to the epidermis. Together, these findings indicate that phyA-mediated effects on phot1 signaling are restricted to low intensities of phototropic stimulation and originate from tissues other than the epidermis.

## Introduction

Since the pioneering work of Charles and Francis Darwin, phototropism has been widely used to investigate how plants alter their growth in response to a directional light stimulus (Christie and Murphy, 2013). Shoot phototropism is typically positive leading to growth toward the light (Briggs, 2014; Liscum et al., 2014; Fankhauser and Christie, 2015), whereas roots frequently show negative phototropism triggering growth away from the light (Kutschera and Briggs, 2012; Mo et al., 2015). Shoot phototropism serves to optimize light capture in low light environments and increase photosynthetic productivity (Fankhauser and Christie, 2015), whereas root phototropism can serve to anchor the root system and provide support for the shoot (Kutschera and Briggs, 2012).

Shoot phototropism in flowering plants is mediated by UV/blue wavelengths of light (Fankhauser and Christie, 2015). Fluence response measurements obtained from a variety of plant species have revealed a surprising degree of complexity for this growth phenomenon (Iino, 2001). Shoot phototropism can be divided into two growth phases depending on the fluence and time requirements. First-positive phototropism is induced by short pulses of light, whereas second-positive curvature (normally observed under natural conditions) occurs in response to prolonged irradiation in a time-dependent manner (Christie and Murphy, 2013). Such properties have been reported for both monocots and dicots (Iino, 2001) suggesting that the mechanisms governing shoot phototropism are conserved in angiosperms.

The role of phototropin (phot1 and phot2) photoreceptors in mediating phototropism is well established (Christie et al., 2015). Phototropins are plasma membrane-associated serine/threonine kinases belonging to the AGCVIII family (Willige and Chory, 2015) that comprise two specialized N-terminal PAS domains, designated LOV1 and LOV2, which serve as UV/blue light sensors by binding the chromophore flavin mononucleotide (Christie et al., 1999). Light sensing is mediated primarily by the LOV2 domain (Christie et al., 2002; Kaiserli et al., 2009) and leads to receptor autophosphorylation (Christie et al., 1998), which is necessary for receptor signaling (Inoue et al., 2008). Phot1 is responsible for mediating second-positive curvature to low fluence rates of blue light in Arabidopsis (≤ 1 μmol m^−2^ s^−1^), whereas phot2 overlaps in function with phot1 to mediate curvature responses at higher fluence rates (Sakai et al., 2001). Thus, phot1 functions as the primary phototropic receptor as it responds to both low and high fluence rates of blue light. Phot1 and phot2 also overlap in function to regulate additional processes that serve to optimize photosynthetic efficiency. These include leaf positioning and expansion, chloroplast relocation movements, and stomatal opening (Christie, 2007; Briggs, 2014).

It has long been proposed that a lateral accumulation of the phytohormone auxin on the shaded side of the stem forms the underlying basis for phototropic growth by promoting differential cell elongation (Sakai and Haga, 2012; Christie and Murphy, 2013; Liscum et al., 2014; Fankhauser and Christie, 2015). The use of auxin response sensors such as *DR5::GFP* and DII-VENUS, whose activities correlate with auxin measurements (Benková et al., 2003; Brunoud et al., 2012), support this model of curvature establishment in Arabidopsis hypocotyls (Christie et al., 2011; Sakai and Haga, 2012; Han et al., 2014; Hohm et al., 2014). Auxin is primarily synthesized in the apical regions where it is actively transported to the roots. This cell to cell movement, referred to as polar auxin transport, is attributed to the action of specific influx and efflux carriers (Spalding, 2013). Arabidopsis mutants lacking the Pin-formed (PIN) auxin efflux carriers PIN3, PIN4, and PIN7 exhibit impaired hypocotyl phototropism in Arabidopsis (Willige et al., 2013) suggesting that multiple transporter proteins are involved in redirecting auxin to the shaded side and initiate curvature toward a phototropic stimulus. However, it is still not known how phototropin activation leads to the changes in auxin trafficking that are required for phototropism.

Non-Phototropic Hypocotyl 3 (NPH3) is a key player in establishing phototropism and acts upstream of auxin redistribution (Liscum et al., 2014). NPH3 interacts with phot1 and has been proposed to regulate auxin redistribution through ubiquitin-mediated proteolysis or re-localization of protein targets (Roberts et al., 2011; Wan et al., 2012). Indeed, NPH3 contains a BTB (Bric-a-brac, Tramtrack and Broad complex) domain, which can function as a substrate-specific adaptor for Cullin-Ring ubiquitin Ligases (Roberts et al., 2011). Root Phototropism 2 (RPT2) is homologous to NPH3 and associates with the phot1-NPH3 complex at the plasma membrane to direct early phototropic signaling events by modulating NPH3 phosphorylation status (Haga et al., 2015). Resolving the biochemical functions of NPH3 and RPT2 and their role in phototropic signaling will be important to understand how lateral auxin gradients are established.

Photoreceptors beside the phototropins are known to modulate phototropism. For instance, pre-exposure of dark-grown (etiolated) seedlings to red light leads to an enhancement of phototropic responsiveness in many plant species including Arabidopsis (Sakai and Haga, 2012; Goyal et al., 2013). This enhancement effect is associated with both first- and second-positive phototropism and is best observed when red light is given 1–2 h prior to the directional blue light stimulus (Haga and Sakai, 2012). Curvature enhancement following red light pre-treatment is dependent on the red/far-red light absorbing photoreceptor phytochrome A (phyA; Janoudi et al., 1997). Photoactivation of phyA in response to red light pre-treatment appears to alter the abundance and action of other AGCVIII kinase family members such as PINOID (PID; Haga et al., 2014). Earlier reports have also indicated that pre-treatment with other wavelengths including white or blue light can lead to phototropic enhancements in etiolated sunflower hypocotyls (Franssen and Bruinsma, 1981). However, their effects on Arabidopsis hypocotyl phototropism have received little attention in recent years. This therefore prompted us to revisit the effects of blue light pre-exposure treatments on second-positive phototropism in Arabidopsis. Additionally, much of the previous data for Arabidopsis hypocotyl phototropism have been obtained from etiolated seedlings grown on vertical agar plates. Studies herein were performed using free-standing seedlings that were grown without the support of an agar surface.

## Materials and methods

### Plant material and growth

Wild-type (*gl-1*, ecotype Columbia), the *phot1-5 phot2-1* and *cry1-304 cry2-1* mutants have been described previously (Mockler et al., 1999; Kagawa et al., 2001). The *phyA-211* (N6223) and *phyB-9* (N6217) single mutants were obtained from the Nottingham Arabidopsis Stock Centre. The *phyA-211 phyB-9* double mutant line was generated by crossing, homozygous plants were identified by genotyping for *phyA-211* (Liu et al., 2001) and the elongated hypocotyl and petiole phenotypes for *phyB-9*. For homozygous lines, the elongated hypocotyl phenotype of the *phyA-211 phyB-9* double mutant was confirmed under red and far-red light. Unless otherwise stated, seeds were planted on soil or surface sterilized and planted on half-strength Murashige and Skoog (MS) medium with 0.8% agar (w/v). After cold treatment (4°C) for 2–4 days, seedlings were grown in a controlled environment room 660 (Fitotron; Weiss-Gallenkamp) under 16 h 22°C:8 h 18°C, light: dark cycles. Fluence rates for all light sources were measured with a Li-250A and quantum sensor (LI-COR).

### Transformation of arabidopsis

Transformation vectors for *PHOT1::PHOT1-GFP* and *ML1::PHOT1-GFP* were constructed using the modified binary expression vector pEZR(K)-LN (Kaiserli et al., 2009). The native *PHOT1* (Preuten et al., 2015; Sullivan et al., 2015) and *ML1* promoters (An et al., 2004) were amplified from Columbia genomic DNA and cloned using the SacI and KpnI restriction sites. The 4.7 kB *PHOT1* promoter was amplified with primers pP1-F (5′-AAAAGAGCTCAAATCAAGAGTTTTGCTTTTCAGG-3′) and pP1-R (5′-AAA AGGTACCTCTCTATACACGAAACAAAAATTGT G-3′) and the 3.4 kB *ML1* promoter was amplified with primers pML1-F (5′-AAC TGAGCTCTTTTACATTGATTCTGAACTGTA CCC-3′) and pML1-R (5′-GATCGGTACCTAGGCTTATAGCCGGTCAAGACA-3′). The full-length coding sequence of *PHOT1* was amplified from cDNA and inserted using the KpnI and BamHI restriction sites. Constructs were transformed into the *phot1-5 phot2-1* double mutant with *Agrobacterium tumefaciens* strain GV3101 as described previously (Davis et al., 2009). Based on the segregation of kanamycin resistance, independent T3 homozygous lines were selected for analysis.

### Phototropism

Seedlings were sowed in transparent plastic entomology boxes (Watkins and Doncaster) on a layer of silicon dioxide (Sigma-Aldrich) watered with quarter-strength MS medium and grown in darkness for 64–70 h. Unilateral blue-light illumination was provided by a white fluorescent tube (GE Lighting; F18W/35) for 0.5 μmol m^−2^ s^−1^ or a slide projector (TLP-T50; Toshiba) for 10 μmol m^−2^ s^−1^ filtered through one layer of blue Plexiglas (Liscum and Briggs, 1995). Images of seedlings were captured every 10 min for 3 h during unilateral illumination with a Retiga 6000 CCD camera (QImaging) connected to a PC running QCapture Pro 7 software (QImaging) with supplemental infrared LED illumination. For pre-treatment with red or blue light, the seedlings were irradiated with overhead white fluorescent light (Philips; TLD 58W/840) filtered through Deep Golden Amber filter No. 135 (Lee Filters) for red light or Moonlight Blue filter No. 183 (Lee Filters) for blue light at a fluence rate of 20 μmol m^−2^ s^−1^ for 15 min. Measurements of hypocotyl angles were made using ImageJ software, version 2.0.0 (http://rsb.info.nih.gov/ij/).

### Chloroplast accumulation

Chloroplast accumulation assays were performed as described previously (Inoue et al., 2011). Rosette leaves detached from 3-week-old plants grown on soil were placed on agar plates and irradiated with 1.5 μmol m^−2^ s^−1^ blue light through a 1-mm slit for 1 h. The plates were placed on a white light trans illuminator and photographed. The blue channel was separated from the resulting RGB image using ImageJ software and presented (Kodama et al., 2010).

### Immunoblot analysis

Total protein extracts were prepared from 3-day-old etiolated seedlings maintained in darkness or irradiated with 20 μmol m^−2^ s^−1^ of blue light for 15 min. Under a dim red safe light, plant tissue was ground in a mortar and pestle in extraction buffer containing 50 mM Tris-HCl pH 7.5, 150 mM NaCl, 1% Triton-X 100, 0.5% sodium deoxycholate, 0.1% SDS, 1 mM phenylmethylsulfonyl fluoride (PMSF) and a protease inhibitor mixture (Complete EDTA-free; Roche) and clarified by centrifugation at 10,000 g, 4°C for 10 min. The resulting supernatant was used as the total protein extract. Protein concentrations were determined by the Bradford colorimetric method (Bio-Rad). All samples were mixed with SDS sample buffer (62.5 mM Tris-HCl, pH 6.8, 2% SDS, 10% glycerol, 5% β-mercaptoethanol, 0.004% bromophenol blue), boiled for 4 min and subjected to 7.5% SDS-PAGE. Proteins were transferred onto polyvinylidene fluoride (PVDF) membrane (GE Healthcare) by electroblotting and detected with anti-phot1 polyclonal antibody (Cho et al., 2007), anti-NPH3 polyclonal antibody (Tsuchida-Mayama et al., 2008), and anti-UGPase polyclonal antibody (Agrisera). Blots were developed with horseradish peroxidase (HRP)-linked secondary antibodies (Promega) and Pierce ECL Plus Western Blotting Substrate (Thermo Fisher Scientific).

### Confocal microscopy

Images of 3-day-old etiolated seedlings expressing GFP-tagged phot1 were visualized using a laser scanning confocal microscope (Zeiss LSM 510) using a C-Apochromat 40X/1.2 water immersion objective. For FM4-64 staining, hypocotyl segments were submerged in 8.2 μM FM4-64 (Sigma) in water for 10 min, rinsed in distilled water and observed immediately. The 488 nm excitation line was used; GFP fluorescence collected with a 505–530 nm band pass filter and FM4-64 fluorescence collected with a 560–615 nm band pass filter. Hypocotyl cross sections were reconstructed from z-stacks using ImageJ software, version 2.0.0 (http://rsb.info.nih.gov/ij/).

### *In vitro* phosphorylation assays

Three-day-old etiolated seedlings were either maintained in darkness or given three different *in vivo* blue-light treatments: 0.5 μmol m^−2^ s^−1^ for 30 min, 20 μmol m^−2^ s^−1^ for 15 min, or 20 μmol m^−2^ s^−1^ for 15 min followed by 0.5 μmol m^−2^ s^−1^ for 30 min. Following each treatment 50 seedlings were collected and immediately frozen in liquid nitrogen. Frozen seedlings were ground directly in 100 μl of kinase buffer containing 37.5 mM Tris-HCl pH 7.5, 5.3 mM MgSO_4_, 150 mM NaCl, 1 mM EGTA, 1 mM DTT and a protease inhibitor mixture (Complete EDTA-free; Roche). Samples were clarified twice by centrifugation at 10,000 g for 15 s and 18 μl of the resulting supernatant used immediately for each phosphorylation assay. Phosphorylation assays were performed as previously described (Christie et al., 2002), in the presence of 1% Triton-X 100. Reactions were performed for 2 min at room temperature and stopped by the addition of sodium dodecyl sulfate (SDS) sample buffer.

## Results

### Immediate pre-treatment with red light accelerates phototropic responsiveness

A modified growth protocol involving silicon dioxide (Sullivan et al., 2015) was used to examine second-positive phototropism in etiolated Arabidopsis. Arabidopsis seedlings readily germinate on silicon oxide moistened with quarter-strength MS medium (Davis et al., 2009). Seed sterilization is not required making the system more convenient compared to growth on MS-agar. To minimize evaporation, etiolated seedlings were grown inside small plastic entomology boxes. These boxes can house ~10 seedlings that grow vertically in the absence of any support. Time-lapse imaging was then used to record the curvature response of seedlings to continuous unilateral blue light treatment at 0.5 μmol m^−2^ s^−1^.

**Figure 1:**
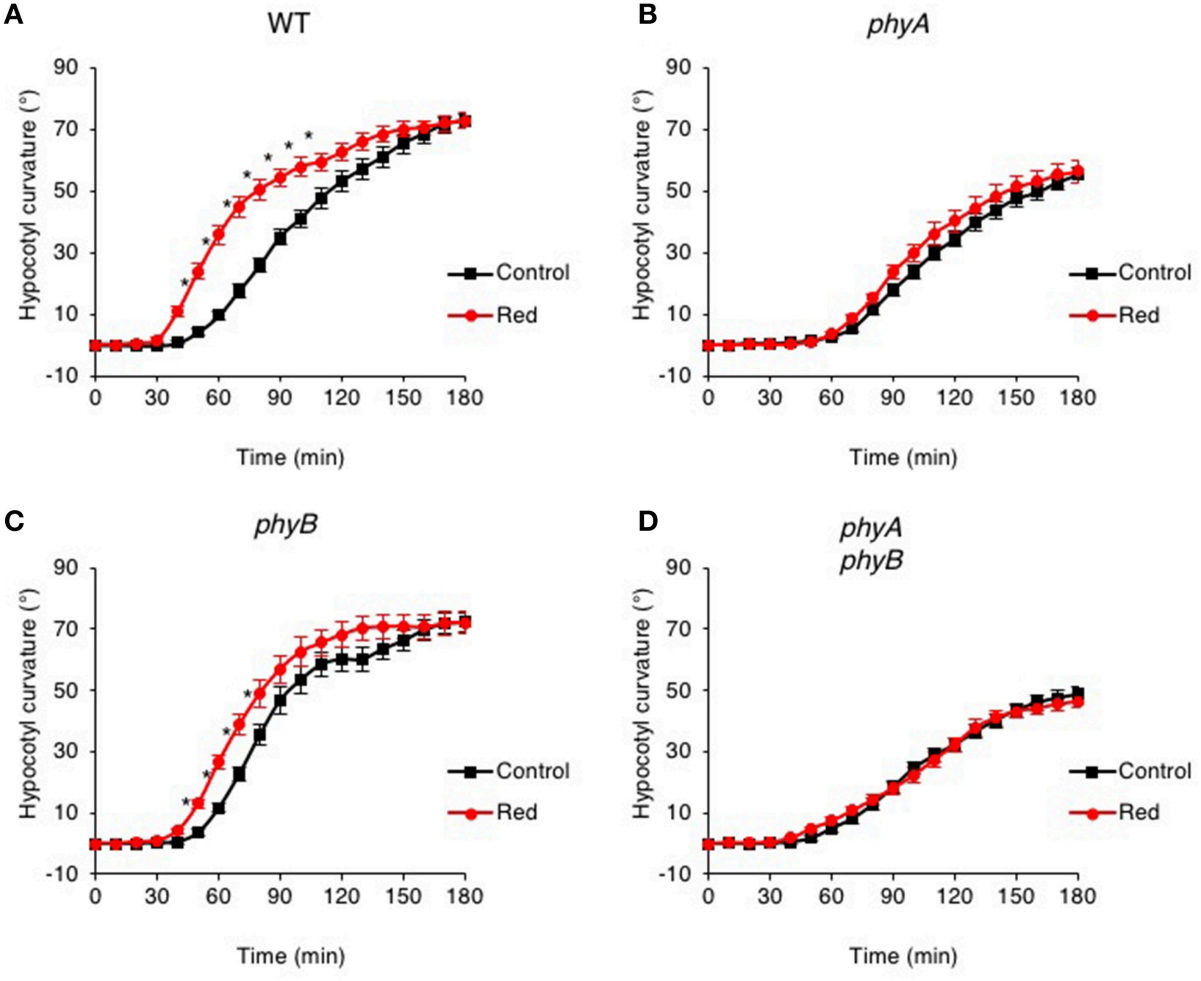
**Pre-irradiation with red light enhances phototropic responsiveness of etiolated seedlings in a phyA-dependent manner**. Three-day-old etiolated WT **(A)** and *phyA* **(B)**, *phyB* **(C)** and *phyA phyB* **(D)** mutant seedlings were maintained in darkness (Control) or irradiated with 20 μmol m^−2^ s^−1^ of over-head red light for 15 min (Red) before being placed into 0.5 μmol m^−2^ s^−1^ of unilateral blue light for 3 h. Hypocotyl curvatures were measured every 10 min and each value is the mean ± S.E. of 18–20 seedlings. Asterisks indicate significant differences between red light treated and control seedlings (*P* < 0.001, Student's *t*-test).

The time course observed for continuous blue light-induced second-positive phototropism of Arabidopsis hypocotyls under these conditions was similar to that previously published for seedlings that are free standing on a horizontal agar surface (Haga et al., 2015), with curvature becoming obvious after 1 h of irradiation and reaching an angle of ~70° after 3 h (Figure 1A). As expected, pre-exposure of seedlings to red light from above (20 μmol m^−2^ s^−1^ for 15 min) enhanced the phototropic responsiveness of etiolated Arabidopsis hypocotyls (Haga and Sakai, 2012; Haga et al., 2014). These studies also demonstrated that red light pre-treatment immediately before phototropic stimulation was sufficient to reduce the lag time for the onset of curvature as reported recently (Haga et al., 2015).

Mutants lacking phyA failed to show enhanced phototropism in response to red light pre-treatment when grown on silicon oxide (Figure 1B). The time course and curvature amplitude for continuous blue light-induced phototropic curvature was also reduced in *phyA* mutants compared to that measured for wild-type etiolated seedlings (Figure 1A). A similar phototropic response was observed in *phyA phyB* double mutants (Figure 1D), whereas *phyB* mutants were still able to show a red light enhancement response (Figure 1C). Together, these findings are consistent with early reports demonstrating that phyA is primarily responsible for the red light enhancement of hypocotyl phototropism in Arabidopsis (Parks et al., 1996; Stowe-Evans et al., 2001).

### Blue light enhances hypocotyl phototropism to low light and is phyA dependent

**Figure 2:**
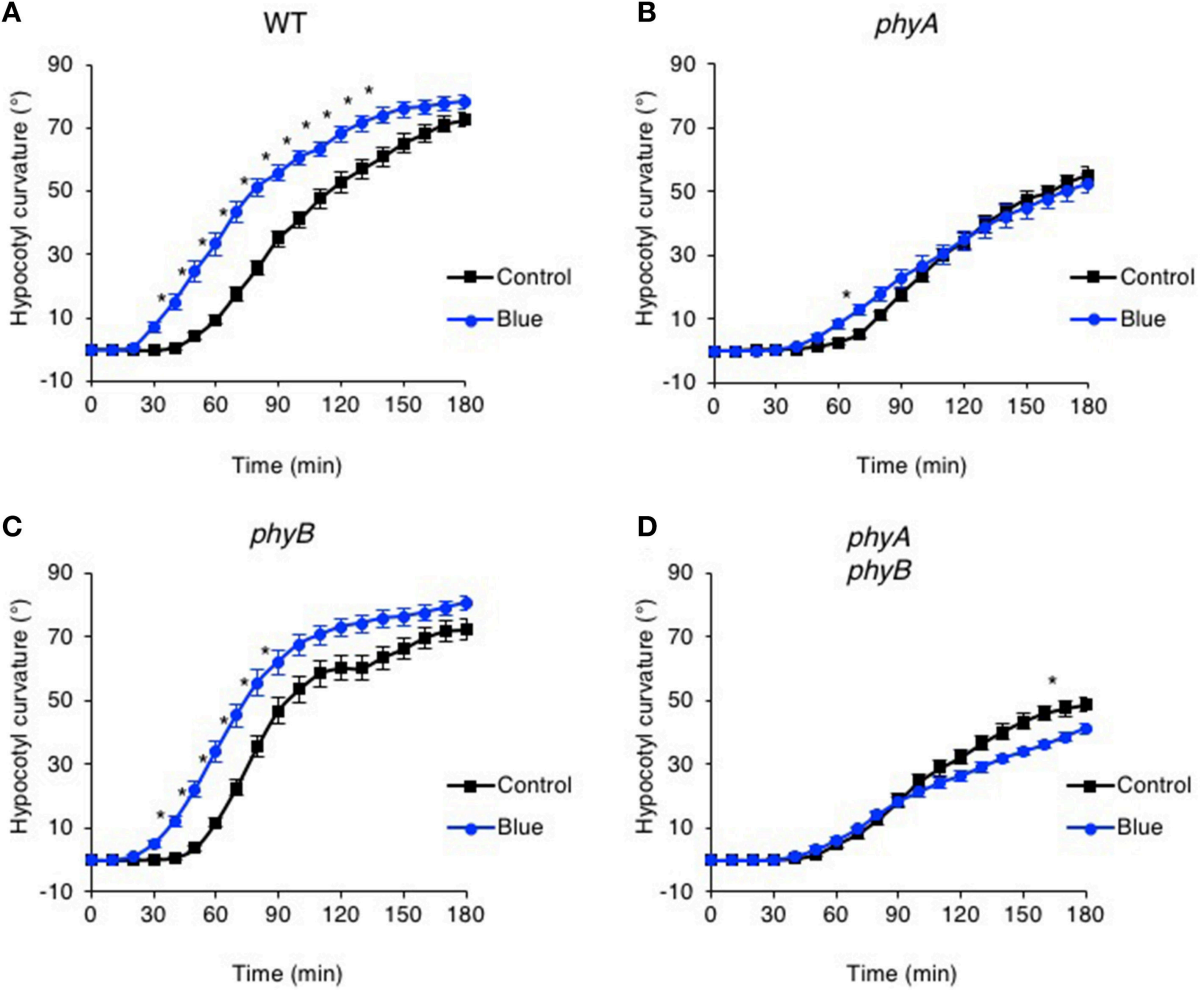
**Pre-irradiation with blue light enhances phototropic responsiveness of etiolated seedlings in a phyA-dependent manner**. Three-day-old etiolated WT **(A)** and *phyA* **(B)**, *phyB* **(C)** and *phyA phyB* **(D)** mutant seedlings were maintained in darkness (Control) or irradiated with 20 μmol m^−2^ s^−1^ of over-head blue light for 15 min (Blue) before being placed into 0.5 μmol m^−2^ s^−1^ of unilateral blue light for 3 h. Hypocotyl curvatures were measured every 10 min and each value is the mean ± S.E. of 17–20 seedlings. Control data as in Figure 1. Asterisks indicate significant differences between blue light treated and control seedlings (*P* < 0.001, Student's *t*-test).

Wavelengths other than red have been reported to impact phototropic responsiveness of etiolated seedlings (Franssen and Bruinsma, 1981). We therefore replaced the red light pre-treatment with blue light to determine whether this could alter the responsiveness of Arabidopsis to subsequent phototropic stimulation. Etiolated seedlings were subjected to a pre-exposure of blue light from above (20 μmol m^−2^ s^−1^ for 15 min) and the effects on hypocotyl curvature to continuous unilateral blue light treatment at 0.5 μmol m^−2^ s^−1^ were recorded by time-lapse imaging. Pre-treatment with blue light was found to be equally as effective as red light in enhancing the phototropic response of Arabidopsis hypocotyls (Figure 2A). Blue light enhancement was still observed in seedlings lacking cryptochrome blue-light receptors cry1 and cry2 indicating that these photoreceptors are not involved (Figure S1). Curvature enhancement in response to blue light pre-treatment was also detected in *phyB* mutants (Figure 2C), but was lacking in the *phyA* and the *phyA phyB* double mutant (Figures 2B,D). Hence, photoactivation of phyA by either red or blue light prior to unilateral blue light irradiation is sufficient to enhance phototropic responsiveness in Arabidopsis hypocotyls.

**Figure 3:**
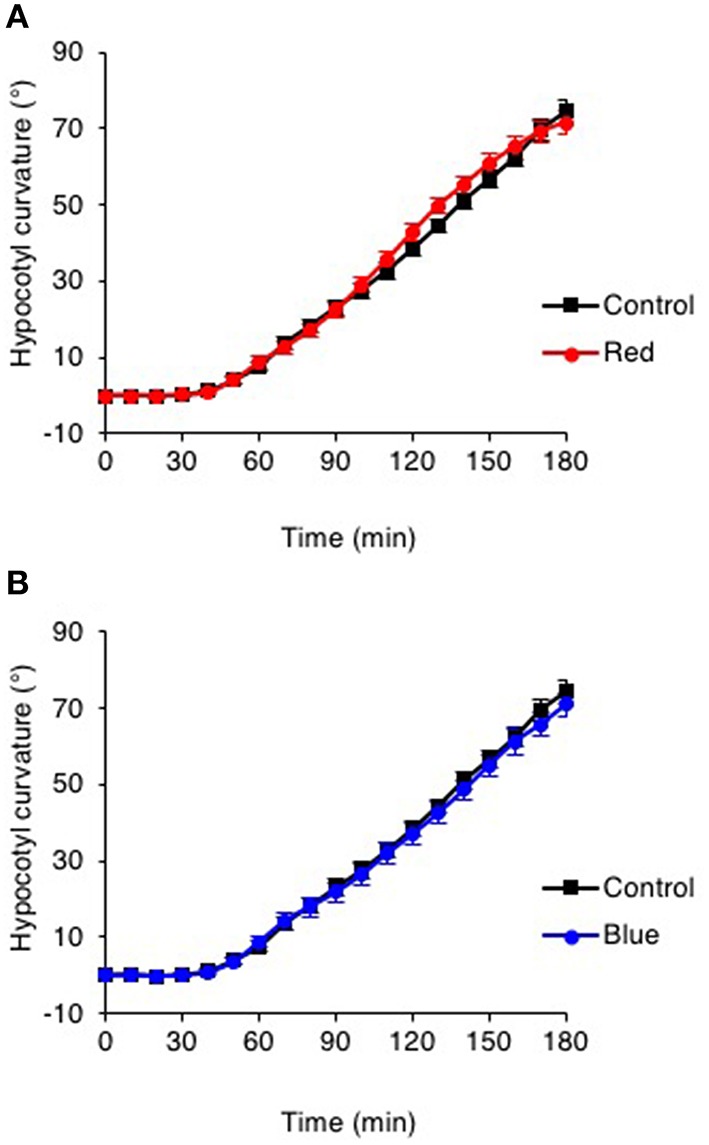
**Pre-irradiation does not enhance phototropic responsiveness to high intensity unilateral blue light. (A)** Three-day-old etiolated WT seedlings were maintained in darkness (Control) or irradiated with 20 μmol m^−2^ s^−1^ of over-head red light for 15 min (Red) or **(B)** irradiated with 20 μmol m^−2^ s^−1^ of over-head blue light for 15 min (Blue) before being placed into 10 μmol m^−2^ s^−1^ of unilateral blue light for 3 h. Hypocotyl curvatures were measured every 10 min and each value is the mean ± S.E. of 20 seedlings.

Phot1 functions to mediate phototropism over a broad range of fluence rates of unilateral blue light (Sakai et al., 2001). We therefore explored whether the effects of red and blue light pre-treatments on hypocotyl phototropism could still be observed following phototropic stimulation at higher intensities of unilateral blue light. While curvature enhancement in response to red and blue light pre-treatments was readily observed at 0.5 μmol m^−2^ s^−1^ (Figures 1A, 2A), this was not the case for seedlings irradiated with 10 μmol m^−2^ s^−1^ unilateral blue light (Figures 3A,B). A blue light pre-treatment was equally ineffective in enhancing phototropism under high intensity unilateral blue light in *cry1 cry2* double mutant seedlings (Figure S2). These findings indicate that the phyA-mediated enhancement effects on hypocotyl phototropism would appear to be restricted to lower intensities of unilateral blue light irradiation.

### Blue pre-treatment induces early phot1 signaling events

**Figure 4:**
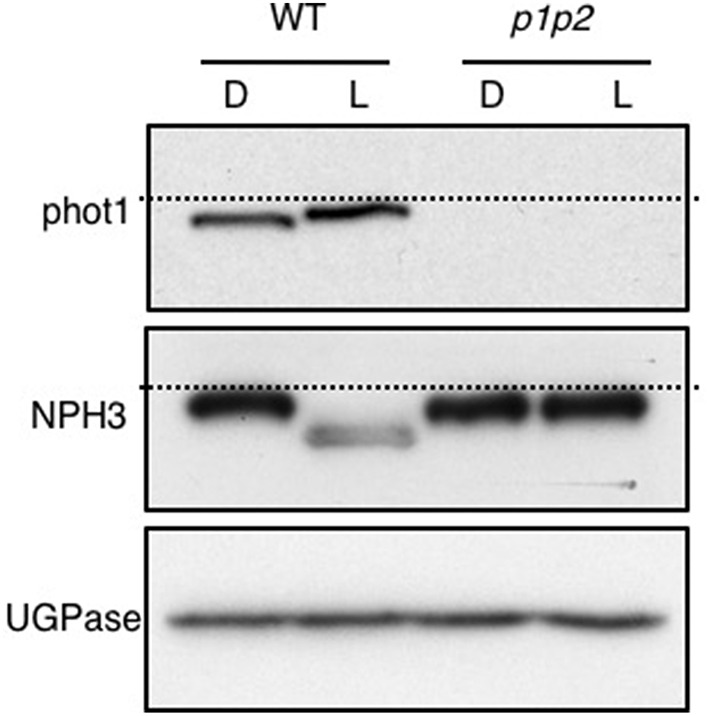
**Blue-light mediated changes in phot1 and NPH3 phosphorylation status**. Immunoblot analysis of total protein extracts from 3-day-old etiolated WT and *phot1 phot2* (p1p2) mutant seedlings maintained in darkness (D) or irradiated with 20 μmol m^−2^ s^−1^ of over-head blue light for 15 min (L). Protein extracts were probed with anti-phot1 (**upper panel**), anti-NPH3 antibodies (**middle panel**) and anti-UGPase antibody as a loading control (**lower panel**). The dashed lines indicate the highest mobility edge.

Unilateral blue light irradiation has been reported to create a gradient of phot1 autophosphorylation across the phototropically sensitive organ (Salomon et al., 1997b). This difference in photoreceptor activation between the irradiated and shaded sides of the stem has been proposed to form the underlying basis for phototropic growth (Salomon et al., 1997b). It was therefore surprising that overhead pre-irradiation with potentially saturating levels of blue light prior to phototropic stimulation did not have an adverse effect on hypocotyl phototropism, but instead resulted in enhanced phototropic responsiveness. Examination of phot1 autophosphorylation status by immunoblotting demonstrated that the blue pre-treatment used in our analysis was sufficient to induce an electrophoretic mobility shift for phot1 (Figure 4), which is characteristic of receptor autophosphorylation and receptor activation (Christie et al., 1998).

The phot1 signaling component Non-Hypocotyl Phototropism 3 (NPH3) is also required for both first- and second-positive phototropism and interacts with phot1 at the plasma membrane (Motchoulski and Liscum, 1999). NPH3 is rapidly dephosphorylated upon phot1 activation (Pedmale and Liscum, 2007; Tsuchida-Mayama et al., 2008) and acts early in the phototropic signaling pathway upstream of auxin redistribution (Haga et al., 2005). We also found that the blue light pre-treatment used in our analysis was sufficient to induce NPH3 dephosphorylation in Arabidopsis seedlings, as measured by its electrophoretic mobility shift (Figure 4). Light-mediated dephosphorylation was not detected in the *phot1 phot2* double mutant. Hence, the blue light pre-treatment employed was able to convert NPH3 to its dephosphorylated state in addition to triggering phot1 autophosphorylation, as monitored by immunoblotting.

### Blue pre-treatment reduces but does not saturate phot1 phosphorylation

Recent work has proposed that the dephosphorylated form of NPH3 is inactive (Haga et al., 2015). It was therefore puzzling that etiolated seedlings were able to retain their ability to respond to a phototropic stimulus following exposure to potentially saturating levels of blue light from above (Figure 2A), especially since NPH3 dephosphorylation and phot1 autophosphorylation were visible by immunoblotting (Figure 4). The phosphorylation status of phot1 in pre-irradiated seedlings was further investigated by subsequently monitoring phot1 kinase activity *in vitro*.

**Figure 5:**
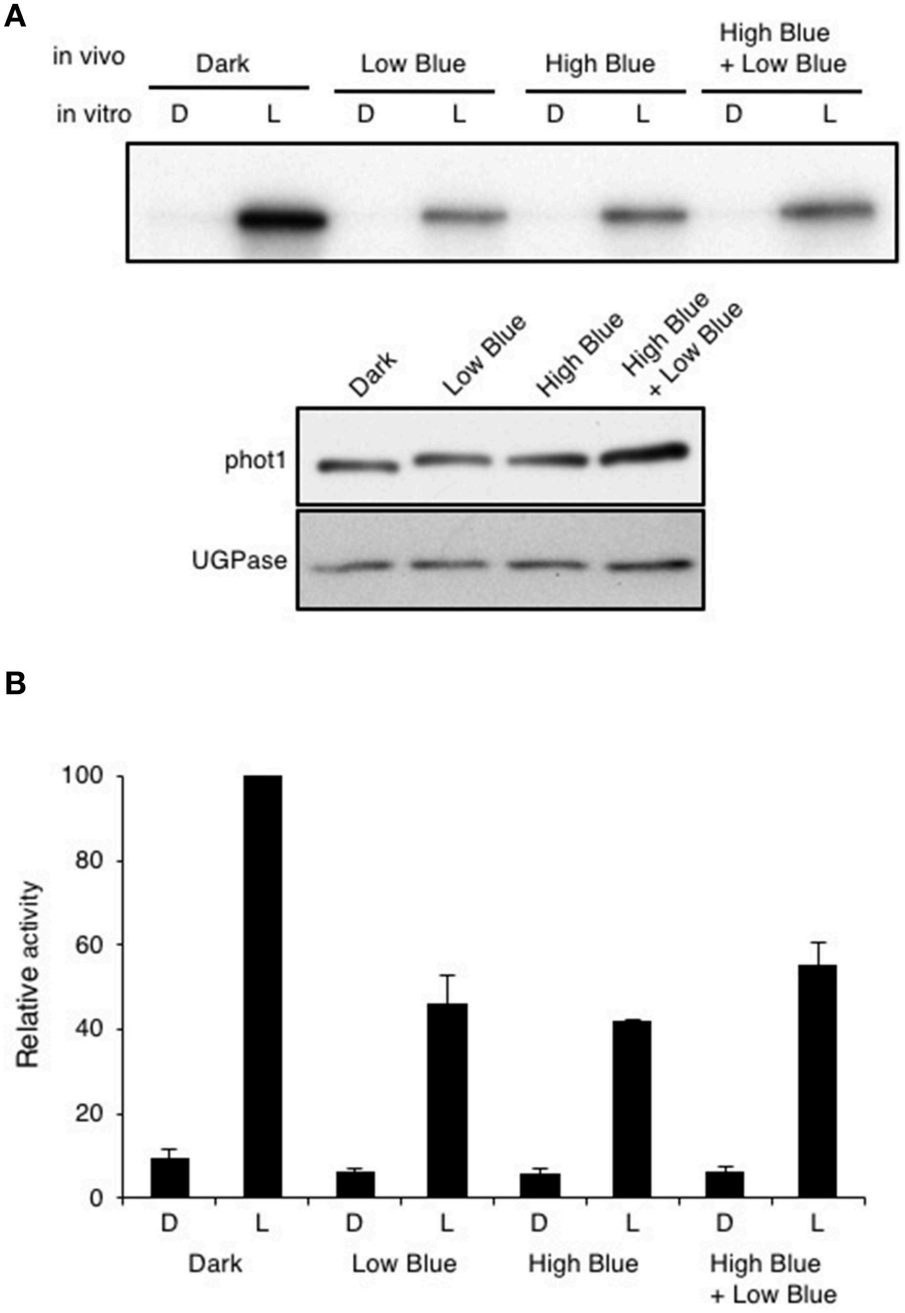
**Effect of *in vivo* blue light irradiation on *in vitro* light-dependent phot1 kinase activity**. Total protein extracts were made from 3-day-old etiolated seedlings maintained in darkness (D) or given one of three different *in vivo* blue-light treatments: 0.5 μmol m^−2^ s^−1^ for 30 min (Low Blue), 20 μmol m^−2^ s^−1^ for 15 min (High Blue), or 20 μmol m^−2^ s^−1^ for 15 min followed by 0.5 μmol m^−2^ s^−1^ for 30 min (High Blue + Low Blue). **(A)** Autoradiograph of *in vitro* kinase assays of total protein extracts given an *in vitro* mock irradiation (D) or irradiated with white light (L) at a total fluence of 30,000 μmol m^−2^ upon addition of radiolabeled ATP. Immunoblot analysis of protein levels with anti-phot1 antibody and anti-UGPase antibody as a loading control is shown in the lower panel. **(B)** Quantification of *in vitro* light-dependent phot1 kinase activity in total protein extracts. Kinase activity was quantified and expressed as a percentage of maximal autophosphorylation.

Autophosphorylation of phot1 is a prerequisite for phototropic signaling (Inoue et al., 2008) and is readily detected when protein extracts isolated from etiolated Arabidopsis seedlings are irradiated *in vitro* with saturating light intensities in the presence of γ-^32^P-labeled ATP (Figure 5; Control). *In vivo* pre-irradiation prior to protein extraction is known to greatly diminish phot1 phosphorylation *in vitro* (Briggs et al., 2001). Similarly, blue light pre-treatment of etiolated Arabidopsis seedlings (Figure 5; High Blue) or irradiation with the same fluence rate of blue light as used for phototropism for 30 min (Figure 5; Low Blue and High Blue + Low Blue) were found to reduce the ability to stimulate phot1 phosphorylation *in vitro* by ~50% (Figure 5). However, a significant level of *in vitro* phosphorylation was still detected following each of the blue light pre-treatments indicating that phot1 autophosphorylation had not been saturated by the *in vivo* irradiation. Detection of phot1 phosphorylation *in vitro* suggests that active phot1 is still present in pre-irradiated seedlings to mediate hypocotyl curvature in response unilateral blue light.

### Curvature enhancement is lacking when phot1 is restricted to the epidermis

To obtain more information regarding the tissue specificity of the molecular events coupling phot1- and phyA-mediated signaling, we generated transgenic Arabidopsis lines expressing phot1 fused to Green Fluorescent Protein (phot1-GFP) in the *phot1 phot2* double mutant under the control of its own promoter as well as the epidermal specific promoter *MERISTEM LAYER 1* (*ML1*). The *PHOT1* promoter is expressed ubiquitously in Arabidopsis seedlings except for the root cap (Sakamoto and Briggs, 2002). However, expression of *ML1::PHOT1-GFP* has been shown previously to fully complement hypocotyl phototropism to continuous lateral blue light in the *phot1 phot2* double mutant of Arabidopsis (Preuten et al., 2013). We therefore investigated whether tissue specific expression of phot1 in the epidermis was also sufficient to restore red and blue light enhancement effects on phototropism.

**Figure 6:**
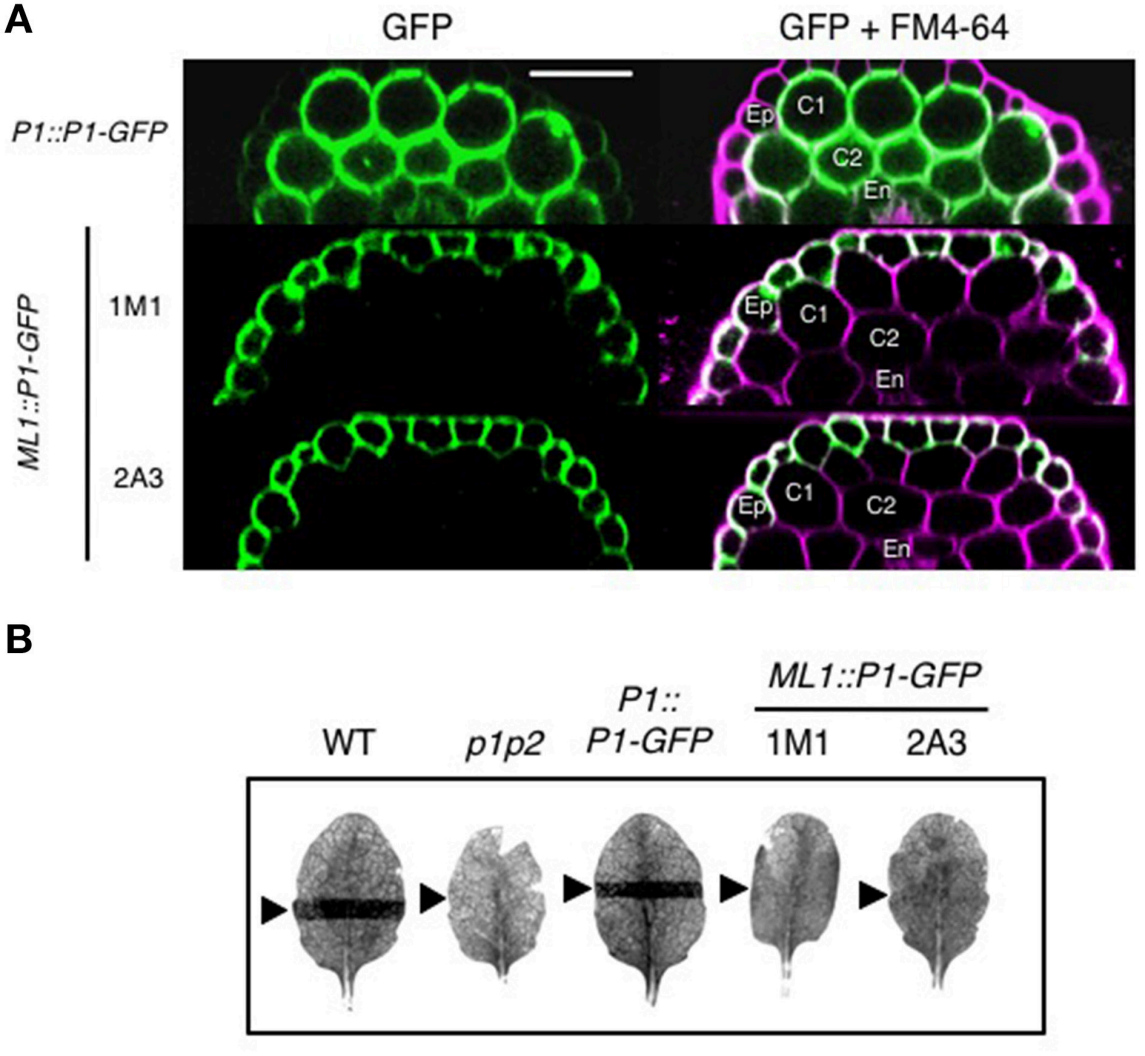
**Epidermal-specific expression of phot1-GFP in *ML1::PHOT1-GFP* transgenic lines. (A)** Phot1-GFP localization in hypocotyls of 3-day-old etiolated seedlings. Reconstructed hypocotyl cross sections of *PHOT1::PHOT1-GFP* (*P1::P1-GFP*) and *ML1::PHOT1-GFP* (*ML1::P1-GFP*) expressing seedlings. FM4-64 staining was used to define cell layers. Ep, epidermis; C1 and C2, cortex layers 1 and 2, respectively; En, endodermis. Scale bar, 50 μm. **(B)** Slit band assays of chloroplast accumulation in wild type (WT), *phot1 phot2* mutants (p1p2) and plants expressing *P1::P1-GFP* and *ML1::P1-GFP*. Detached leaves were irradiation with 1.5 μmol m^−2^ s^−1^ of blue light through a 1 mm slit for 60 min. Arrows indicate irradiated areas.

To verify tissue specific expression, we monitored the cellular distribution of phot1-GFP in the hypocotyls of the transgenic lines generated. Under the control of the *PHOT1* promoter (Sullivan et al., 2015), phot1-GFP was detected predominantly in the hypocotyl cortex and endodermis of etiolated seedlings and to a much lesser extent in the epidermis (Figure 6A). By contrast, *ML1::PHOT1-GFP* expression was only visible in the hypocotyl epidermis of independent T3 homozygous lines confirming tissue specific expression (1M1 and 2A3; Figure 6A). To further ensure the tissue specificity of *ML1::PHOT1-GFP* expression, we first examined the ability of these lines to mediate chloroplast accumulation movement, since expression of phot1 in the epidermis would not be expected to complement this response in the mesophyll.

Chloroplast accumulation movement was assessed using the slit band assay (Kagawa et al., 2001; Suetsugu et al., 2005). When leaves of wild-type or *PHOT1::PHOT1-GFP* expressing plants were irradiated with 1.5 μmol m^−2^ s^−1^ of blue light through a 1 mm slit, a dark band was visible on the leaf due to a chloroplast accumulation response (Figure 6B). No band was visible in the *phot1 phot2* double mutant owing to the lack of these photoreceptors. Likewise, both *ML1::PHOT1-GFP* expressing lines failed to restore chloroplast accumulation movement giving us confidence that the localization observed in these lines was specific to the epidermis.

**Figure 7:**
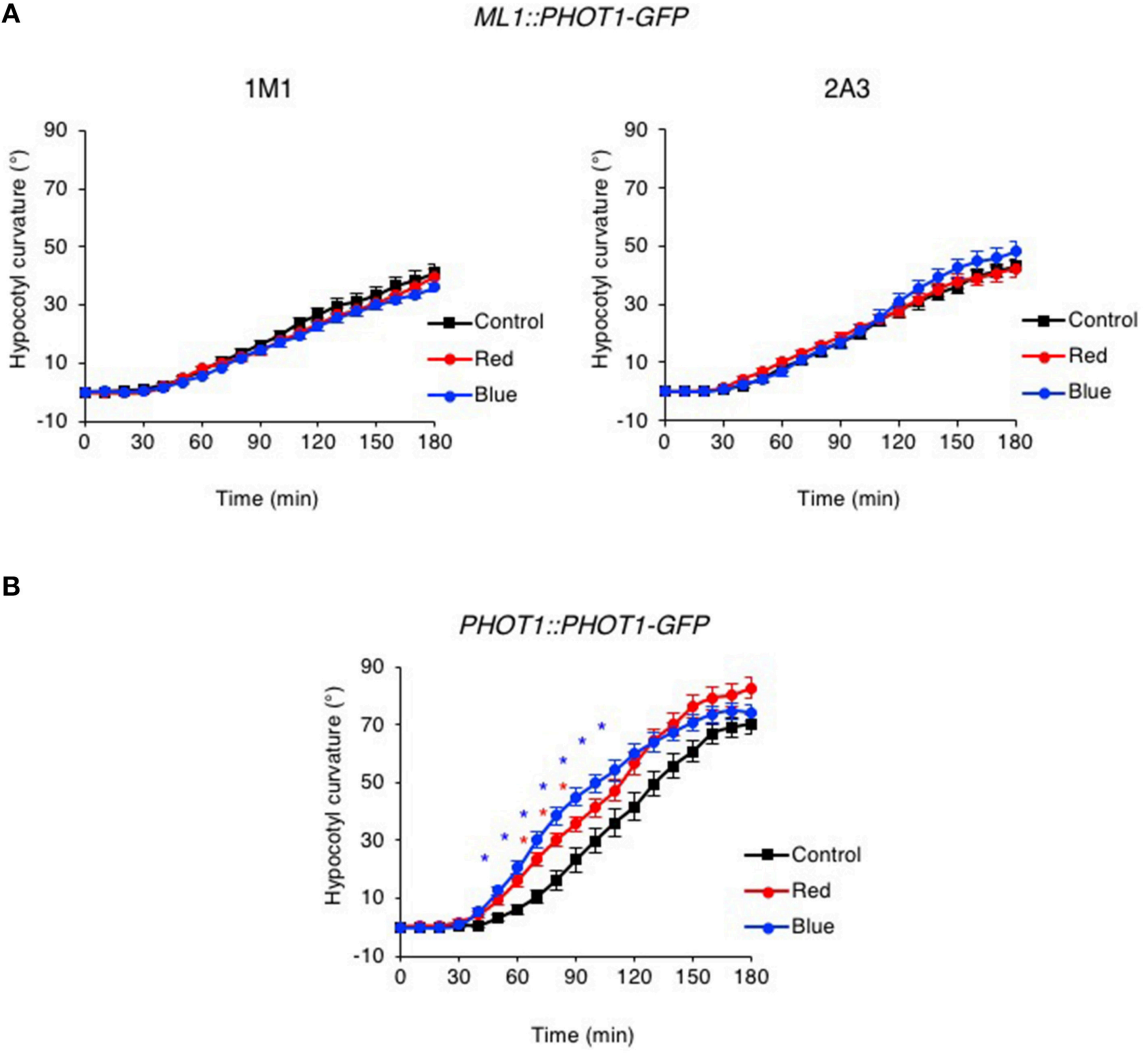
**Pre-irradiation does not enhance phototropic responsiveness in *ML1::PHOT1-GFP* transgenic lines**. Three-day-old etiolated seedlings expressing *ML1::PHOT1-GFP* **(A)** or *PHOT1::PHOT1-GFP* **(B)** were maintained in darkness (Control), irradiated with 20 μmol m^−2^ s^−1^ of over-head red light for 15 min (Red) or irradiated with 20 μmol m^−2^ s^−1^ of over-head blue light for 15 min (Blue) before being placed into 0.5 μmol m^−2^ s^−1^ of unilateral blue light for 3 h. Hypocotyl curvatures were measured every 10 min and each value is the mean ± S.E. of 16–21 seedlings. Asterisks indicate significant differences between red or blue light treated and control seedlings (*P* < 0.001, Student's *t*-test).

While no chloroplast accumulation movement was detected in the *ML1::PHOT1-GFP* expressing lines, hypocotyl phototropism was observed in response to continuous lateral blue light irradiation at 0.5 μmol m^−2^ s^−1^ (Figures 7A,B) consistent with previous reports (Preuten et al., 2013). However, the time course and amplitude of the response in *ML1::PHOT1-GFP* expressing lines closely resembled those of *phyA* mutants (Figure 1B). Furthermore, no phototropic enhancement in response to either a red or blue light pre-treatment was observed for the *ML1::PHOT1-GFP* expressing lines (Figure 7A), despite being detected in *PHOT1::PHOT1-GFP* expressing seedlings (Figure 7B). In our hands, *ML1::PHOT1-GFP* expressing lines (Figure 7A) appear to phenocopy the phototropic responsiveness of *phyA* mutants (Figure 1B). These findings therefore indicate that restriction of phot1 to the epidermis impairs the ability of phyA to mediate its enhancement effects on second-positive hypocotyl phototropism in Arabidopsis.

## Discussion

Much of the early work on first-positive phototropism has focused on using grass coleoptiles as a model system. Such studies have shown that pre-treatment with high intensities of bilateral blue light immediately prior to unilateral photo stimulation is sufficient to stop the onset of first-positive phototropism in maize coleoptiles (Iino, 1987). A reduction in first-positive phototropism by bilateral pre-irradiation with blue light has also been reported for Arabidopsis seedlings (Janoudi and Poff, 1991). However, introducing dark intervals between these light treatments could restore phototropic responsiveness. A similar refractory mechanism has also been reported for phot1 phosphorylation following a saturating blue light pulse both in maize (Palmer et al., 1993) and oat coleoptiles (Salomon et al., 1997a). *In vivo* irradiation promotes phot1 phosphorylation and subsequently reduces the ability to detect phot1 kinase activity *in vitro* in isolated membrane extracts (Briggs et al., 2001). However, phot1 phosphorylation *in vitro* can be regained if membranes are isolated following increasing dark periods given after the *vivo* irradiation. Together, these findings suggest that the phot1-mediated first-positive phototropism is rapidly desensitized upon high intensity illumination and subsequently resensitized following a refractory period of several minutes, as had been proposed by Briggs over half a century ago (Briggs, 1960).

Results presented in this study however, demonstrate that overhead pre-irradiation of etiolated Arabidopsis seedlings with blue light immediately prior to phototropic stimulation results in an enhancement of second-positive phototropism. This was unexpected considering that phot1 autophosphorylation and dephosphorylation of the phototropic signaling component NPH3 were detected by immunoblotting following the pre-irradiation treatment (Figure 4). Autophosphorylation of phot1 within the kinase activation loop is required for phototropism in Arabidopsis (Inoue et al., 2008). NPH3 in its dephosphorylated state was recently proposed to represent the inactive form of the protein (Haga et al., 2015). With this in mind, we initially expected pre-treated seedlings to exhibit some refractory period for the photosensory system to recover before being able to respond to phototropic stimulation. Instead, hypocotyl phototropism in response to continuous unilateral blue light irradiation at 0.5 μmol m^−2^ s^−1^ was enhanced by the blue pre-treatment (Figure 2A).

Active phot1 was still observed in pre-treated seedlings as measured by the ability to detect phot1 autophosphorylation in protein extracts isolated after blue light pre-treatment (Figure 5). We therefore conclude that the light conditions used for our phototropic measurements were not sufficient to saturate phot1 activation *in vivo*. Instead, these experiments indicate the availability of light-activateable photoreceptor within blue light pre-treated seedlings that would account for their phototropic responsiveness despite being given blue light prior to phototropic stimulation. It is however worth noting that the fluences used to saturate phot1 phosphorylation *in vitro* are typically one to two orders of magnitude higher than those used to saturate first-positive phototropism (Briggs et al., 2001). The reason for this discrepancy is still not fully understood, but illustrates that there are limitations to consider when correlating receptor phosphorylation with phototropic responsiveness. Nevertheless, pre-irradiation with blue light (20 μmol m^−2^ s^−1^ for 15 min, equating to a total fluence of 18,000 μmol m^−2^) is unable to impair second-positive hypocotyl phototropism in Arabidopsis to 0.5 μmol m^−2^ s^−1^ unilateral blue light, but instead leads to an enhancement of phototropic responsiveness (Figure 2), as was observed when a red pre-treatment was used (Figure 1).

In addition to red and far-red light, blue light is known to activate phyA and modulate phototropism (Lariguet and Fankhauser, 2004). Enhancement of second-positive phototropism to red and blue pre-irradiation was absent in the *phyA* mutant (Figures 1B, 2B) demonstrating that phyA is required for both these processes. As well as lacking curvature enhancement to pre-irradiation, *phyA* mutants also exhibited reduced phototropism to 0.5 μmol m^−2^ s^−1^ unilateral blue light when compared to wild type. These findings are consistent with those of Whippo and Hangarter (2004) showing that phyA activity is necessary for the progression of normal hypocotyl phototropism in Arabidopsis to low fluence rates of lateral blue light, as monitored by time-lapse imaging (Whippo and Hangarter, 2004). Given that phot1 is the directional light sensor mediating phototropism under these conditions (Sakai et al., 2001), phyA signaling therefore appears to modulate phot1 action both during phototropic stimulation at low light intensities, as well as in response to pre-irradiation treatments.

The molecular mechanisms by which phyA and phot1 signaling are integrated still remain poorly understood. Although the action of phyA on phototropism has been attributed to an attenuation of blue light-induced internalization of phot1 from the plasma membrane (Wan et al., 2012), curvature enhancements in response to red pre-treatments are still observed in seedlings where phot1 has been anchored to the plasma membrane by lipid modification (Preuten et al., 2015). It has also been proposed that phyA exerts its action on phototropic signaling from the cytosol (Rösler et al., 2007). However, constitutive targeting of phyA to the nucleus is sufficient to promote its effect on phototropism in Arabidopsis (Kami et al., 2012). Therefore, phyA-mediated gene regulation of key signaling components is likely to be involved. For example, expression of the AGCVIII kinase PID is down-regulated in etiolated Arabidopsis seedlings following a red light pre-treatment and appears to have a role in attenuating first- and second-positive phototropism together with other PID family members (Haga et al., 2014).

The data presented in this study demonstrate that expression of *ML1::PHOT1-GFP* was sufficient to restore hypocotyl phototropism in Arabidopsis in the *phot1 phot2* double mutant to low intensities of lateral blue light (Figure 7) as was shown previously (Preuten et al., 2013). However, the phototropic responsiveness of these transgenic lines combined with the lack of curvature enhancement in response to either a red or blue light pre-treatment lead us to conclude that phyA exerts its effect on phot1 signaling outside the epidermis. These findings are in agreement with a recent study by Kirchenbauer et al. (in press) showing that expression of *ML1::PHYA-YFP* is unable to promote phototropism in the *phyA* mutant in response to 1 μmol m^−2^ s^−1^ unilateral blue light irradiation following a far-red light pre-treatment (Kirchenbauer et al., in press). By contrast, expression of *PHYA-YFP* under control of the mesophyll specific promoter *CHLOROPHYLL A/B BINDING PROTEIN 3* (*CAB3*) was found to partially restore phyA action on hypocotyl curvature leading the authors to propose that phyA localized mainly within the cortical cells of the hook region plays an important role in regulating blue light induced phototropism. Transgenic lines expressing *CAB3::PHOT1-GFP* would be expected to show similar effects if the mechanisms integrating phyA and phot1 signaling are tissue autonomous. Indeed, initial studies indicate that *CAB3::PHOT1-mCitrine* expression in the *phot1 phot2* double restores wild-type level of second-positive phototropism to 1 μmol m^−2^ s^−1^ unilateral blue light (Preuten et al., 2013). Phototropism was also fully restored in transgenic lines expressing *PHOT1-mCitrine* under the control of the *ML1* promoter, an endodermis specific promoter or a cortex specific promoter. However, the ability of these tissue-specific promoters to drive phot1 functionality in processes other than phototropism was not examined in this study. Further examination of such lines with respect to phot1-mediated responses such as leaf expansion, chloroplast accumulation and stomatal opening would provide more information regarding the stringency of tissue-specific expression.

Our analysis also found that Arabidopsis seedlings lacked curvature enhancements in response to red or blue light pre-treatments when higher intensities of unilateral blue light were used for phototropic stimulation (Figure 3). Whippo and Hangarter (2003) have previously shown, by time-lapse imaging, that the kinetics and magnitude of phototropic curvature is reduced at increasing blue light intensities of phototropic stimulation (Whippo and Hangarter, 2003). The authors concluded that the reduction in the phototropic response observed under high fluence rates of blue light (100 μmol m^−2^ s^−1^) is caused by an inhibition of hypocotyl elongation mediated through the activities of both cryptochrome and phototropin blue-light receptors. Mutants lacking phot1, phot2, or cry1 and cry2 showed enhanced phototropic responsiveness when irradiated with 100 μmol m^−2^ s^−1^ unilateral blue light implying that the activity of these photoreceptors act to attenuate the curvature response under high light conditions. Our studies indicate that phototropic stimulation at 10 μmol m^−2^ s^−1^ impacts phyA signaling since no curvature enhancements were observed in WT seedlings in response to red or blue light pre-irradiation (Figure 3), or in seedlings lacking cry1 and cry2 in response to blue light pre-irradiation (Figure S2). The mechanism(s) that act to antagonize phyA action under these light conditions requires further investigation.

The results presented in this study provide new insights into the action of phyA in modulating phototropic responsiveness of etiolated Arabidopsis seedlings. In contrast to angiosperms, phototropism in fern species including Adiantum capillis-veneris is mediated by the chimeric phytochrome-phototropin receptor neochrome 1 (neo1) (Kawai et al., 2003). The responsiveness to both red and blue wavelengths serves to ensure increased photosensitivity in shaded habitats. Expression of neo1 in the phototropin-deficient mutant of Arabidopsis has been shown to result in phototropic responsiveness to red light in addition to blue (Kanegae and Kimura, 2015). A better understanding of the interplay between photoreceptor systems in model organisms such as Arabidopsis will help elucidate how flowering plants have evolved different levels of complexity in controlling phototropism. In this regard, further examination of Phytochrome Kinase Substrate (PKS) proteins will be of particular interest given their known role in phototropin signaling (Demarsy et al., 2012).

## Author contributions

JC and SS designed and directed the research. SS, JH, PR, and CW planned and performed experiments. All authors analyzed aspects of the data. JC and SS wrote the manuscript.

### Conflict of interest statement

The authors declare that the research was conducted in the absence of any commercial or financial relationships that could be construed as a potential conflict of interest.

## Supplementary material

Figure S1 **Pre-irradiation with red or blue light enhances phototropic responsiveness of etiolated *cry1 cry2* seedlings. (A)** Three-day-old etiolated *cry1 cry2* mutant seedlings were maintained in darkness (Control) or irradiated with 20 μmol m^−2^ s^−1^ of over-head red light for 15 min (Red) or **(B)** irradiated with 20 μmol m^−2^ s^−1^ of over-head blue light for 15 min (Blue) before being placed into 0.5 μmol m^−2^ s^−1^ of unilateral blue light for 3 h. Hypocotyl curvatures were measured every 10 min and each value is the mean ± S.E. of 20 seedlings. Asterisks indicate significant differences between red or blue light treated and control seedlings (*P* < 0.001, Student's *t*-test).

Figure S2 **Pre-irradiation does not enhance phototropic responsiveness of *cry1 cry2* seedlings to high intensity unilateral blue light**. Three-day-old etiolated *cry1 cry2* mutant seedlings were maintained in darkness (Control) or irradiated with 20 μmol m^−2^ s^−1^ of over-head blue light for 15 min (Blue) before being placed into 10 μmol m^−2^ s^−1^ of unilateral blue light for 3 h. Hypocotyl curvatures were measured every 10 min and each value is the mean ± S.E. of 20 seedlings.
