# Supplementary figures and images for: Phytochrome A Mediates Blue-Light Enhancement of Second-Positive Phototropism in Arabidopsis
Source: Front Plant Sci. 2016 Mar 11;7:290. doi: 10.3389/fpls.2016.00290 (PMC4786545; doi:10.3389/fpls.2016.00290)

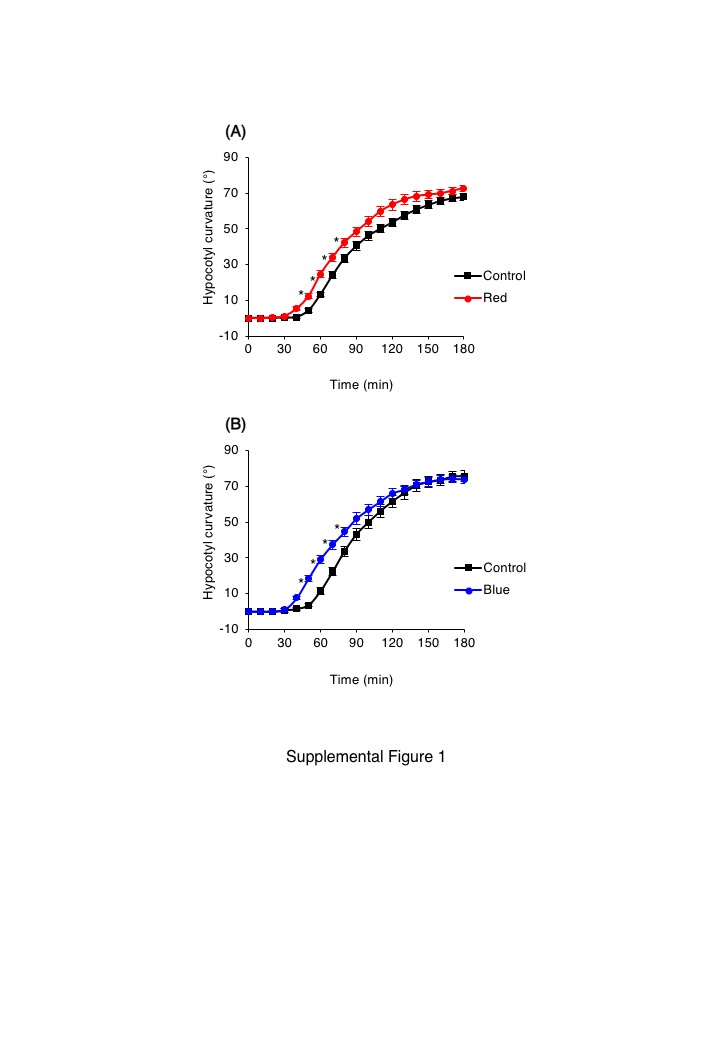

Supplement: Figure S1 — Pre-irradiation with red or blue light enhances phototropic responsiveness of etiolated cry1 cry2 seedlings. (A) Three-day-old etiolated cry1 cry2 mutant seedlings were maintained in darkness (Control) or irradiated with 20 μmol m−2 s−1 of over-head red light for 15 min (Red) or (B) irradiated with 20 μmol m−2 s−1 of over-head blue light for 15 min (Blue) before being placed into 0.5 μmol m−2 s−1 of unilateral blue light for 3 h. Hypocotyl curvatures were measured every 10 min and each value is the mean ± S.E. of 20 seedlings. Asterisks indicate significant differences between red or blue light treated and control seedlings (P < 0.001, Student's t-test). [file Image1.JPEG]

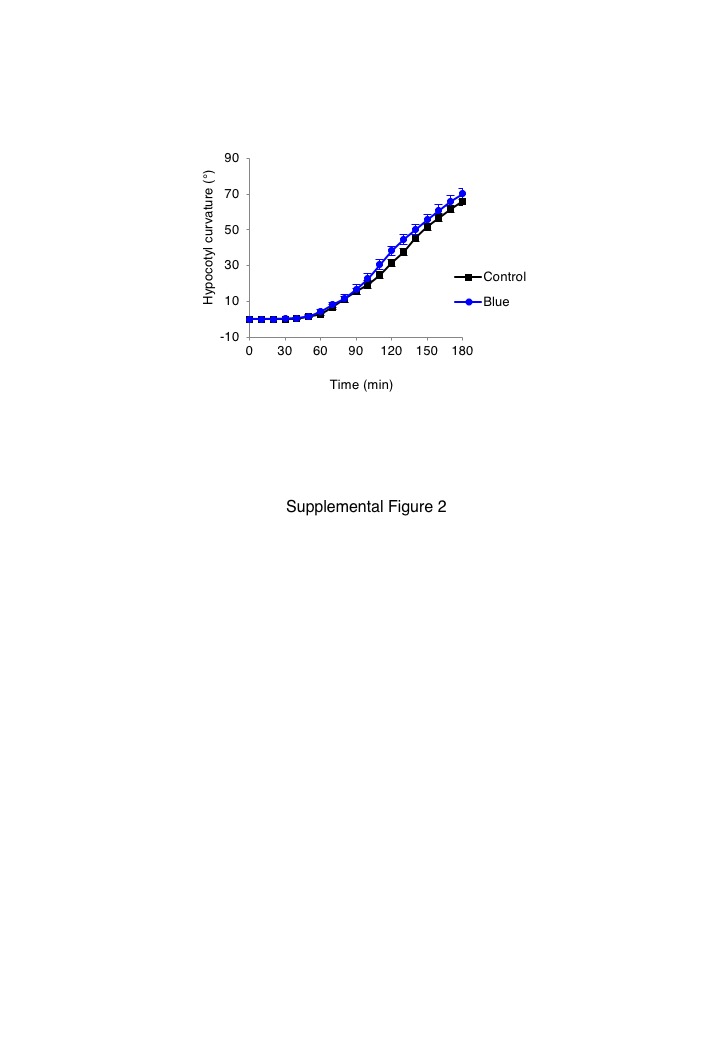

Supplement: Figure S2 — Pre-irradiation does not enhance phototropic responsiveness of cry1 cry2 seedlings to high intensity unilateral blue light. Three-day-old etiolated cry1 cry2 mutant seedlings were maintained in darkness (Control) or irradiated with 20 μmol m−2 s−1 of over-head blue light for 15 min (Blue) before being placed into 10 μmol m−2 s−1 of unilateral blue light for 3 h. Hypocotyl curvatures were measured every 10 min and each value is the mean ± S.E. of 20 seedlings. [file Image2.JPEG]
